# Lack of Adherence to Isoniazid Chemoprophylaxis in Children in Contact with Adults with Tuberculosis in Southern Ethiopia

**DOI:** 10.1371/journal.pone.0026452

**Published:** 2011-11-01

**Authors:** Kefyalew T. Garie, Mohammed A. Yassin, Luis E. Cuevas

**Affiliations:** 1 College of Medicine and Health Sciences, Hawassa University, Hawassa, Ethiopia; 2 Liverpool School of Tropical Medicine, Liverpool, United Kingdom; 3 Global Fund to Fight AIDS, Tuberculosis and Malaria, Geneva, Switzerland; McGill University, Canada

## Abstract

**Setting:**

Hawassa, Southern Region of Ethiopia.

**Objectives:**

To determine compliance to isoniazid (INH) preventive therapy (IPT) and its effectiveness in preventing (TB) disease in children in contact with adults with pulmonary TB (PTB).

**Design:**

This was a prospective cohort study of children <15 years old in contact with adults with smear-positive PTB. Asymptomatic children ≤5 years were provided IPT independently of their Tuberculin Skin Test (TST) status and children >5 years old were given advice but did not receive IPT, as recommended by the National TB control programme. Compliance to IPT and incidence of clinical TB were determined monthly for six months and then quarterly for up to 30 months.

**Results:**

One hundred and eighty four children in contact with 83 smear-positive PTB cases were identified. Eighty two were ≤5 and 102>5 years old. Only 27 (33%) of 82 children given IPT took it for >4 months and 10 (12%) completed the 6-month course. The main reason for non-compliance was the perception that drugs were not necessary when the child was healthy. Eleven children (all except one >5 years old) developed symptoms of TB disease and initiated treatment, resulting in an incidence of 28.6 cases for all and 53.5 for children >5 years old per 1000 children-year.

**Conclusion:**

Compliance to IPT in children is poor in Southern Region of Ethiopia and this was associated with the parents' perception of the low importance of chemoprophylaxis in asymptomatic children. Poor compliance might be an important barrier for the wider implementation of IPT.

**Trial Registration:**

Clinicaltrials.gov NCT00456469

## Introduction

Tuberculosis (TB) is one of the leading causes of morbidity and mortality globally (WHO, 2009) and children are estimated to represent between 13% and 15% of cases in high TB burden countries (HBC) [1]. TB is one of the five major causes of death in Ethiopia (WHO, 2006) and in the Southern Nations, Nationalities and People's Region (SNNPR) childhood TB represents 7.7% of new smear-positive and 13% of all forms of TB reported [2].

Young children have a high risk of TB infection and disease progression because of their intimate contact with adults with pulmonary TB (PTB) [3] and immature immune system [4]. Isoniazid (INH) Preventive Therapy (IPT) has been shown to prevent new infections and inhibit progression from infection to TB disease and in HBC IPT can reduce the incidence by around 40% among Tuberculin Skin Test (TST)-positive individuals [5]. It is thus recommended that children <5 years old in contact with adults with smear-positive PTB are screened for tuberculosis disease, and if no disease is found, offered IPT [6]. TB control programmes however rarely have the resources and manpower for implementation and several studies have reported that adherence to IPT is generally poor [7], [8], [9].

There is no information on patterns of adherence to IPT and the incidence of TB among children in contact with adults with PTB in Ethiopia. This study therefore aimed to determine the level of IPT adherence of children in contact with adults with smear-positive PTB in the SNNPR and the incidence of TB among children receiving and not receiving IPT.

## Methods

The study was approved by the SNNPR Health Bureau, the Research Ethics Committees of Hawassa University and the Liverpool School of Tropical Medicine and the National Ethics Review Council of Ethiopia.

This was a prospective cohort study and was based in the SNNPR, one of the largest regions of the country with over 15 million population. Consecutive adults diagnosed as having smear-positive PTB and who lived with children <15 years old were invited to participate when attending Bushullo Major, Hawassa Health centre and Hawassa University Hospital in Hawassa, the capital of the SNNPR. After obtaining informed consent, families were visited to document demographic and socio-economic characteristics of the family and enter the household location into a Geographical Positioning System to facilitate the identification of households during the follow up. All children were examined and laboratory tests were conducted as needed to exclude TB disease. Three children had symptoms compatible with TB and were referred for diagnosis. These children were then diagnosed as having TB, treated with anti-TB drugs and excluded from the cohort presented here. As part of the cohort study, TST was applied to all asymptomatic children to describe the natural course of potential markers of infection over time (including TST, Interferon-γ and IP10 and others, data not shown). TST was applied with Purified Protein Derivative (Serum Statens Institute, Denmark) equivalent to 2 International Units of Tuberculin on the volar aspect of arm using the Mantoux method and according to the manufacturer's instructions and TST readings were obtained 48–72 hours later using the palpation method. TST indurations were classified as negative (<5 mm), intermediate (5–9 mm) and positive (≥10 mm) [10]. Blood samples were collected and tested in duplicate for HIV using two ELISA methods.

All children <5 years old were provided INH (5 mg/kg/day) for 6 months, independently of the TST results, while older children underwent the same examinations and testing but were not given IPT, according to the National TB Control Programme guidelines [11]. All children were enrolled during a period of one year and followed for between 17 and 30 months (the maximum duration of the study). Children were followed monthly for the first 6 months and then every three months for up to a maximum of 30 months. Parents were informed about the need for IPT and to report any adverse effect, the importance of adherence to treatment and to attend the study clinics if the child developed symptoms of ill health suggestive of TB between the visits. INH tablets were supplied free of charge in blister packets during the monthly visits and parents were asked whether their child had experienced adverse effects or developed symptoms since the previous visit. IPT compliance was assessed by asking parents if the child had taken the pills, checking the blister packs and counting pills. Parents who had not given the pills to the child were asked the reasons for the lack of compliance, were reminded of the advantages of IPT, and advised to resume the pills. IPT compliance was classified as reasonable, poor or very poor (if taken for more than 4 months, 2 to 4 or <2 months, respectively) [12]. Children with symptoms compatible with TB were referred to a pediatrician for further clinical, laboratory and radiological investigations and to initiate treatment, as required.

Data were entered into an SPSS database and checked for discordances after data cleaning. P values<0.05 were considered statistically significant.

## Results

One hundred and eighty four children in contact with 83 adults with smear-positive PTB were identified and followed between October 1, 2007 and April 30, 2009. Of these, 88 (47.8%) were male and 36% resided in rural areas. On enrolment their age ranged from 1 to 15 years and 82 (45%) were <5 years old. One hundred and six (58%) children had positive, 22 (12%) intermediate and 56 (30%) negative TST. The majority of children <5 years old had negative or intermediate TST, while most children >5 years old had positive TST, as shown in table 1. Sixteen (10.8%) of 148 children tested for HIV were positive.

**Table 1:** Characteristics of children who received and not received INH prophylaxis.

Characteristics on enrolment		≤5 years old (N = 82)	>5 years old (N = 102)
Male (%):		39 (47.5)	50 (49)
Mean (SD) age (years):		2.9 (1.4)	9.9 (3)
Weight for height Z score	<−3SD	5 (6.1)	1(0.9)
	−3SD to −2SD	3 (3.7)	10 (9.8)
	>−2SD	74 (90.2)	91 (89.3)
Contact time per day (mean (SD), hours)		11.6 (5.7)	8.1 (4.2)
TST result	positive	38 (46%)	68 (67%)
	intermediate	14 (17%)	26 (25%)
	negative	30 (47%)	8 (8%)
BCG scar present, N (%):		52 (63.4)	58 (56.9)

N = number, SD, Standard deviation, mm = millimeter, TST = Tuberculin skin test.

The median follow up duration was 24 months with a range of 17 to 30 months. All parents of children <5 years old agreed to give IPT to their children. TST indurations of children with and without IPT were different, with children receiving IPT having smaller indurations than children not receiving IPT. On follow up, there were no adverse effects reported among children receiving IPT. Only 27 (32.9%) children had reasonable IPT compliance, while 24 (29.3%) had poor and 31 (37.8%) very poor compliance. Among children with reasonable compliance, only 10 (12.2% of 82) completed the 6-month IPT course. Thirty seven (67%) of 55 parents whose children had poor/very poor compliance indicated the main reason for non-compliance was the perception that drugs were not necessary when the child was healthy.

Eleven (6%) children were diagnosed as having TB disease during the follow up period, resulting in an incidence of 28.6 cases per 1000 person-years follow up for all children and 53.5 for children age >5 years old. The characteristics of these 11 children who developed TB disease are shown in table 2. Their mean age on enrolment was 9.2 years, which was slightly lower than children who did not develop symptoms (p = 0.9, not significant) and all but one (91%) had a positive TST, compared to only 96 (55.5%) of 172 children without TB. Two children had smear-positive PTB, seven were diagnosed as having smear-negative PTB and two had cervical TB lymphadenitis. The median time from enrolment to diagnosis of TB was 120 days (range 3 to 403 days). Children who develop TB were less likely to live in households with windows and none of them had received IPT, as shown in Table 3, although the number of children with TB was small and the comparison needs to be interpreted with caution.

**Table 2:** Characteristics of children who developed tuberculosis disease (N = 11).

Age* (years)	Sex	TST (mm)	HIV	BCG scar	Adult smear grade	Window**	Treatment outcome	Presentation
2	Male	22	Neg	Yes	1+	No	Completed	SS^−^PTB
6	Female	19	Neg	Yes	1+	No	Completed	EPTB
6	Female	19	Neg	No	scanty	No	Completed	EPTB
6	Male	6	Neg	Yes	2+	No	Completed	SS^+^PTB
7	Male	18	Neg	Yes	1+	No	Completed	SS^−^PTB
10	Female	13	Neg	Yes	2+	No	Completed	SS^−^PTB
10	Male	21	NA	Yes	1+	No	Completed	SS^−^PTB
12	Male	21	Neg	Yes	1+	Yes	Completed	SS^−^PTB
14	Female	19	Neg	Yes	1+	No	On treatment	SS^+^PTB
14	Male	19	Neg	Yes	1+	Yes	Completed	SS−PTB
14	Male	19	Neg	Yes	1+	No	Completed	SS^−^PTB

*At the time of enrolment.

**Presence of windows in the home; NA = Not available. EPTB = Extrapulmonary TB, SS+ PTB = Smear positive pulmonary TB, SS− PTB = Smear negative pulmonary TB, Neg = negative, TST = Tuberculin skin test.

**Table 3:** Characteristics of children with and without TB disease among those followed.

Characteristics		TB (N = 11)	No TB (N = 173)	P-value
Male		7 (64%)	81(47%)	0.3
Mean (SD) age (enrolment)		9.2 (4%)	6.7 (4%)	0.9
TST* (mm)	Positive	10 (92%)	96 (56%)	0.02
	Intermediate	1 (9%)	56 (32%)	
	Negative	0 (0%)	21 (12%)	
HIV	Positive	0 (0%)	16 (9%)	0.3
	Negative	10 (91%)	122 (71%)	
	Unknown	1 (9%)	35 (20%)	
Smear grade of the adult*				
	Scanty	1 (9%)	3 (2%)	0.15
	+	8 (73%)	115 (67%)	
	++	2 (18%)	41 (24%)	
	+++	0 (0%)	14 (8%)	
Home has windows		2 (18%)	119 (69%)	0.001

*Chi square for trend, N = number, TB = tuberculosis, mm = millimeter, SD = Standard deviation.

## Discussion

Children, and especially young children, in contact with adults with PTB are at high risk of infection due to their intimate and prolonged contact with adult index cases within the household. The TST positivity rate among the children enrolled in our study was high, but within the range reported from similar settings (e.g. 63% in Zimbabwe [13] and 49% in Nigeria [14]. The TST positivity rate in children in contact with adults with TB was much higher than the rate observed in children without known exposure to TB in the same area (13%, [15]). Young children have a high risk of progression from infection to disseminated disease because their immunological system is unable to contain and localize the bacilli, often resulting in severe forms of TB such as miliary TB and meningitis. Nearly all TB Control programmes worldwide recognize this increased risk and support the notion of providing prophylaxis for young children.

Despite this awareness, guidelines are rarely implemented in resource-poor settings due to a lack of resources. Reports of IPT uptake and adherence are scarce and poorly documented, with only a few scientific reports reporting IPT adherence in the last decade. Within the African context, two studies in South Africa reported that IPT adherence was 20% in a retrospective review of records and 27.6% in a prospective cohort of children exposed to adults with PTB [9], [12]. In Malawi, only 17% of children participating in a cohort with passive follow up completed 6 months of IPT compared to 22% for children with active follow up [16]. These findings suggest that the low IPT adherence observed in our study is unlikely to be unusual and that further studies are needed to identify approaches that facilitate adherence in the African continent.

The most frequent reason for the poor adherence was that parents did not perceive IPT as an important method to reduce the risk of disease progression in the absence of symptoms. The same reasoning was reported in South Africa [12], suggesting that interventions to increase adherence need to address the population's perception that drugs are not needed for asymptomatic individuals. Key factors to ensure the success of IPT programmes therefore are to make the drugs and the services available regularly, a better understanding of the parents' perception of the risk of disease after exposure, their awareness of the importance of adherence in the absence of symptoms, the development of effective education and communication packages to convey these messages and addressing the concerns of health staff in the community.

A significant proportion of children in the study were HIV-positive. The risk of disease progression in general is highest in immunocompromised children with HIV co-infection and studies to document the effectiveness and scaling-up feasibility of IPT in HIV-infected individuals are currently under way. IPT however is already recommended for HIV-infected individuals in many countries and understanding its acceptability and likely adherence under operational conditions, and the parents and staff perception of the need of IPT and risk of drug resistance, together with a description of the constraints faced by health systems would be important to facilitate implementation.

None of the children receiving IPT developed TB disease, while 6% of older children not receiving IPT were diagnosed with TB disease during the follow up period, although only a small proportion of them were microbiologically confirmed. None of the children who developed TB disease were co-infected with HIV, suggesting that the risk of disease progression in older children is not negligible even without HIV co-infection. The estimated incidence of all forms of TB in the general population in Ethiopia in 2009 was 359 per 100,000 [17] and the incidence of TB disease among the children followed (28.6 per 1000 children-year) was very high. This finding highlights the need for contact investigation and providing IPT and allocating adequate resources to prevent TB in contacts of infectious TB cases. Although for operational and resource constrains older children are often excluded, IPT may be of benefit for this age group in high TB incidence settings.

This study therefore illustrates that compliance to IPT in young children is poor in Southern Region of Ethiopia and that the lack of compliance is associated with the parents' perception of the importance of chemoprophylaxis. The reluctance of parents to resume IPT after counseling indicates that this might be an important barrier for its wider implementation and further studies are needed to develop communication strategies to effectively convey these messages.
